# PNIPAM/Hexakis as a thermosensitive drug delivery system for biomedical and pharmaceutical applications

**DOI:** 10.1038/s41598-022-18459-3

**Published:** 2022-08-23

**Authors:** Samaneh Pasban, Heidar Raissi

**Affiliations:** Department of Chemistry, University of Birjand, Birjand, Iran

**Keywords:** Biotechnology, Cancer, Drug discovery, Molecular biology, Systems biology, Diseases, Rheumatology, Nanoscience and technology

## Abstract

Many technologies ranging from drug delivery approaches to tissue engineering purposes are beginning to benefit from the unique ability of “smart polymers.” As a special case, thermo-sensitive hydrogels have great potential, e.g. in actuators, microfluidics, sensors, or drug delivery systems. Here, the loading of Doxorubicin (DOX) with novel thermo-sensitive polymer N-isopropyl acrylamide (PNIPAM) and its copolymers are investigated in order to increase the Doxorubicin’s drug efficacy on the targeted tumor site. Therefore, a rational design accurate based on the use of classical molecular dynamics (MD) and well-tempered metadynamics simulations allows for predicting and understanding the behavior of thermo-responsive polymers in the loading of DOX on Hexakis nano-channel at 298 and 320 K. Furthermore, this work investigates the efficacy of this drug carrier for the release of DOX in response to stimuli like variations in temperature and changes in the physiological pH. The study concludes that the Hexakis–polymer composite is capable of adsorbing the DOX at neutral pH and by increasing the temperature of the simulated systems from 298 to 320 K, the strength of intermolecular attraction decreases. In addition, the obtained results of MD simulation revealed that the dominant interaction between DOX and Hexakis in the DOX/polymer/Hexakis systems is the Lennard–Jones (LJ) term due to the formation of strong π–π interaction between the adsorbate and substrate surface. Obtained results show that a higher aggregation of DMA chains around the Hexakis and the formation of stronger bonds with DOX. The results of the well-tempered metadynamics simulations revealed that the order of insertion of drug and polymer into the system is a determining factor on the fate of the adsorption/desorption process. Overall, our results explain the temperature-dependent behavior of the PNIPAM polymers and the suitability of the polymer–Hexakis carrier for Doxorubicin delivery.

## Introduction

Poly(N-isopropylacrylamide) (PNIPAM) polymer, is one of the most widely used and thoroughly studied temperature-sensitive polymers^1–4^. The temperature response of polymers is important for both the fundamental understanding of polymer physics and technical applications^5,6^. PNIPAM polymer exhibits a lower critical solution temperature (LCST) approximately at 310 K (37 °C)^7,8^. It is completely soluble in the water below LCST and becomes less soluble or even collapses in the water phase above LCST. Since this LCST is near to the temperature at which most physiological processes happen, it makes PNIPAM a promising material for the development of targeted drug delivery systems^9–11^. Regarding cancer treatment, PNIPAM polymer is generally applied to study the interaction between biological cognition and target cells at different temperatures or pH^12–14^. To understand the LCST behavior of these polymers, as well as the effect of the polymer chain on drug loading, drug release, cell delivery, several studies have been performed by researchers. Tucker and Stevens^15^ studied the polymer chain length (over the range of 3–30 mer) depending on the transition temperature for a single syndiotactic PNIPAM polymer and found the existence of higher LCST for shorter chains. Reza Maleki et al.^16^ investigated the effect of thermo-sensitive N-isopropyl acrylamide polymer chain length on the carbon nanotube as a drug delivery system for the loading of Doxorubicin (DOX) via classical molecular dynamics (MD) simulations. Their obtained results revealed the PNIPAM with chain length, i.e., 15 mer is more stable and effective in delivery systems than the higher chain length PNIPAM polymer based on the energetics and structure of the system. Vatti et al.^17^ via molecular dynamics simulations, studied the solubility of the Doxorubicin in three different polymers, i.e., poly (N-isopropyl acrylamide), polyethylene glycol, and polyvinyl pyrrolidone. Their work suggested that the PNIPAM 15-mer length is the most stable and effective in drug delivery systems. Using the theoretical studies, Murti et al.^18^ reported that PNIPAM-grafted graphene oxide (GO) creates an “on”/“off” surface-state around its LCST in interactions with the cancer-cell protein. In fact, the presence of the PNIPAM monomer stabilizes the system due to the interaction between the nucleobases and GO. Besides, Shiddiky and co-workers^19^ have produced PNIPAM polymer-based immunosensors that provide a reversible surface for recognizing cancer protein in human serum. More recently, Aleman and co-workers^20^ have developed Semi-interpenetrated nanogels (NGs) consisting of a poly (N-PNIPAM) and dendritic polyglycerol (dPG) mesh containing a SIPN (semi-interpenetrating network). Their results revealed that PNIPAM polymer provides thermos-responsivity and acts as a stabilizer. In addition, the collapse of PNIPAM polymer increases the contact between the intrinsically conducting polymers (ICPs) chains, enhances the charge transfer processes, and facilitates the interaction with the electrode surface. Besides many studies on pure PNIPAM polymer, investigations on its co-polymers have also been performed and revealed interesting insights^21–25^. Among the different ring-like macrocycles, Hexakis (m-PE) structures with rigid backbones and non-deformable cavities are of particular interest^26,27^. These structures consist of oligo-(**m-**phenylene ethylene) units with strictly defined inner and outer diameters, which are commonly known as useful biocompatible materials^28^. In our previous studies^29,30^, for the first time through MD simulations and density functional theory (DFT) calculations, it has been shown that Hexakis (m-PE) undergo a self-assembly process to form a nanotubular structure that can be a novel biocompatible sensor for drug delivery systems. Doxorubicin, serving as an anticancer drug model, is one of the reliable conventional chemotherapy drugs^31,32^. Many studies have shown that the usage of nanocarriers for the delivery of chemotherapeutic drug DOX as adjuvant therapy reduces the toxicity of this agent and increases its effectiveness^33–35^. Additionally, the development of new therapeutic strategies for the non-toxicity and selective delivery of DOX to tumors is of great importance. Compared to the conventional drug delivery systems (DDS), the advantages of the smart DDS are self-evident. Nanocomposites are of great interest in nanobiotechnology due to their competency to show good multifunctional properties. Different types of nanocomposites have been successfully developed to date, and each model can be used for different applications. On the other hand, the nanocomposite can be used for the controlled release of drugs, and a combination of them with nanoparticles can be promising tools for targeted drug delivery. For example, Gupta et al.^36^ work on the synthesis and applications of the polysorbate/ironmolybdophosphate (PS/FMP) nanocomposite. In addition, the PS/FMP nanocomposite is used as a drug delivery vehicle for targeted or systemic delivery of methylcobalamin drugs. Their obtained results show that the drug encapsulation efficiency and drug loading efficiency are found to be about 35.2% and 60.4%, respectively. The release of methylcobalamin is found to be pH 9.4 > pH 7.4 > saline (pH 5.7) > pH 2.2. Based on their results, the PS/FMP nanocomposite is a promising multifunctional nanocomposite. Hence, in the present study, classical (MD) and well-tempered metadynamics simulations are performed to study Hexakis–PNIPAM nanocomposite as a suitable carrier for DOX delivery. In order to study the effect of temperature dependence profiles of polymers on PNIPAM characteristics and drug delivery, two different temperatures are considered for PNIPAM and two of its co-polymers, and six simulations are done with the loading of DOX along with 10-mer polymers on the surface of Hexakis nanotube. In addition, the DOX release mechanism from the DMA/Hexakis, DMA as the most stable polymer, at acidic conditions has been evaluated. For this purpose, PNIPAM oligomer and two of its copolymers, namely NIPAAm-codimethylacrylamide (p(NIPAAm-co-DMA)), (here is shortly called DMA), and pNIPAAm-co-acrylamide (p(NIPAAm-co-Am)), (here is shortly called Am), are used at 298 and 320 K. For analysis of this attractive carrier, the interaction energies, gyration radius, radial distribution function, and free energy landscape are studied. The results of this study can provide a good and novel insight into the optimal use of PNIPAM polymers for controlled release of DOX drug. Since the tumor environment has a higher temperature than the rest of the body, temperature-sensitive polymers can generally release drugs at that point. By studying the Hexakis–polymer as a carrier, this question is answered: do Hexakis–polymer serve as a suitable candidate for the controlled release of DOX and drug release following temperature increase in cancer tumors?

## Computational methods and simulation details

### Preparation of starting models

**Scheme 1:**
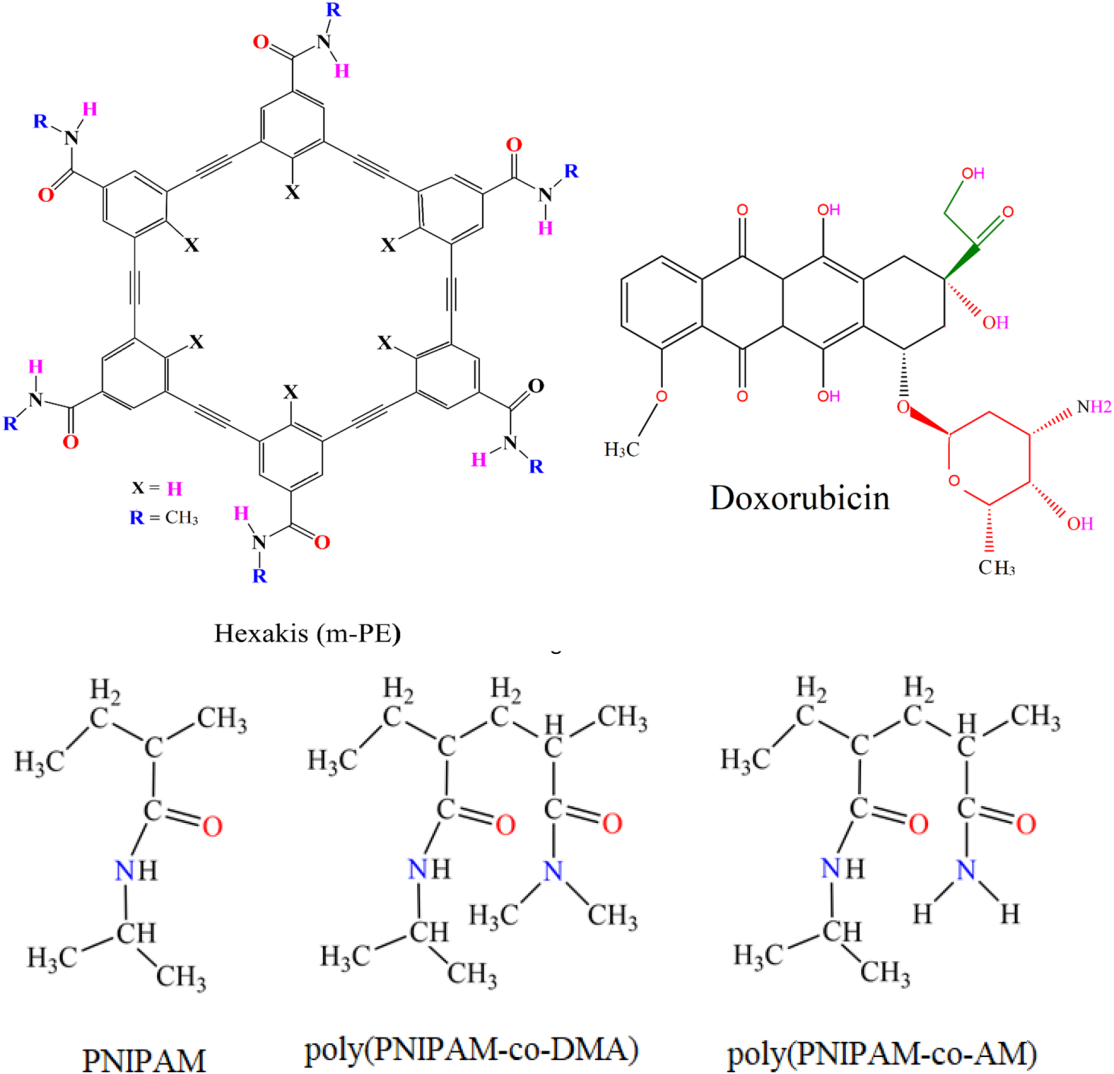
The representations of the polymer chains, DOX molecule, and Hexakis ring.

**Figure 1:**
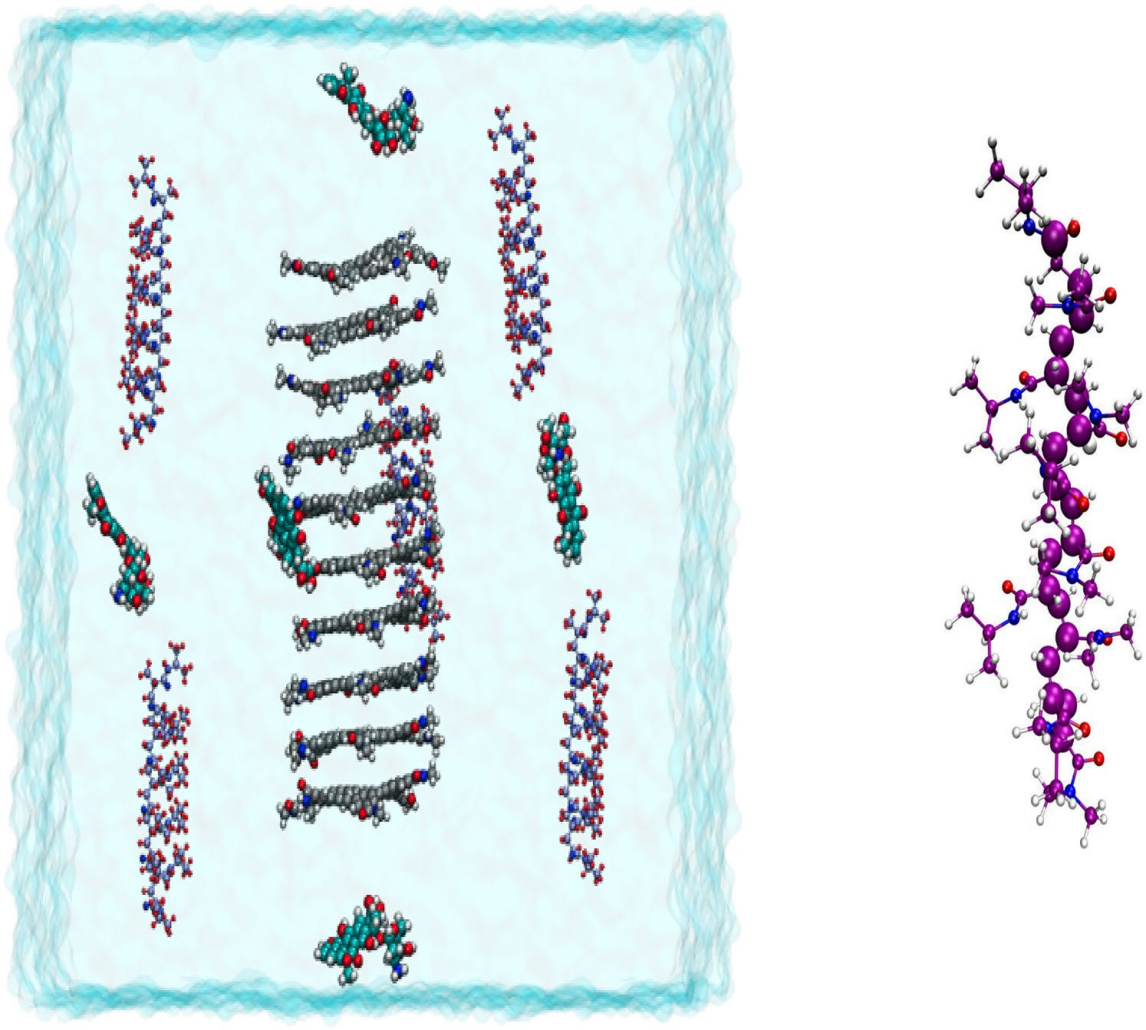
Left: The initial snapshots of DOX–Polymer–Hexakis systems. Color code DOX: vdW cyan, Polymer: CPK violet, Hexakis: silver. The ions molecules are not shown for clarity. Right: Snapshot of DMA polymer chains for 10-mer (backbone carbon atoms of CH2 are shown in violet).

The initial structure of the Hexakis nanotube is obtained from the final equilibrium simulations of our previous simulation study^29^, which contains ten rings of Hexakis (m-PE) macrocycles. The PNIPAM polymer and two of its co-polymers chains (each chain contains ten repeat monomers) are built using Gauss view software^37^ and optimized at the DFT level of theory (m062x/6–31G ∗)^38–40^ using the Gaussian09 package program^41^. The M06-2X approach have been developed by Truhlar and coworker that this method covers the dispersion energy missing at the DFT level by a damped pair potential empirical term. it can be stated that the M06-2X functional can be used as an appropriate method for assessing the accuracy of the geometry relaxations and related parameters. In fact, the M06-2X functional can be able to perform both electrostatic and dispersion interactions. Further, the partial atomic charges for the PNIPAM and its co-polymer are calculated via fitting the electrostatic potential using the CHELPG method as implemented in the Gaussian09 code. The results of the optimized structures are utilized in the MD simulations. Scheme 1 shows the 2-D representations of the polymer chains, DOX molecule, and Hexakis ring. To evaluate the PNIPAM and its copolymer's effects on the adsorption of DOX molecules on the Hexakis surface, three simulation systems are considered as follows: (PNIPAM/DOX/Hexakis) system A, (Am/DOX/Hexakis) system B, and (DMA/DOX/Hexakis) system C. However, these systems are investigated at temperatures below (298 K) and above (320 K) the LCST. In addition, the “release” mechanism of the drug molecules from the carrier surface, at acidic pH to mimic tumor environmental pH, is examined. For the construction of this system, the final configuration of the DOX–DMA–Hexakis complex is given from the loading system at 298 K and then all of the amine groups of DOX and DMA are protonated (PDMA/PDOX/Hexakis) system D). Furthermore, it is worthy to mention that the protonation degree for poly(PNIPAM-co-DMA) polymer that is used to mimic pH changes is about 49.97%. In each system, a certain number of polymer chains and DOX molecules with different charge fractions are located approximately 20 Å away from the surface of the Hexakis nanotube. This distance is chosen to avoid the effect of initial configurations. The simulation boxes are chosen to be sufficiently large to prevent any bias of the Periodic Boundary Conditions. The simulation trajectories are visualized using the Visual Molecular Dynamics (VMD) program^42^. Figure 1 shows a sample of the initial simulation box (before each simulation run).

### MD simulation details

All MD simulations in this work are performed with the GROMACS simulation package^43^. Force-field parameters for DOX, Hexakis, and polymers are taken from the CHARMM force field^44^. The TIP3P model is used for water molecules. For reproducing the correct biological environment and neutralizing the studied systems, 0.15 M sodium chloride is added toto the simulation boxes. First, the energy in each system is minimized using the steepest descent algorithm to remove any bad contacts in the system. In the next stage, each system is gradually increased to 310 K in 500 ps under the NVT conditions and equilibrated at 310 K for another 1000 ps at constant pressure p = 1 bar under NPT conditions. For these two equilibration runs, the systems are coupled using a Berendsen thermostat. Finally, production runs for 60 ns MD simulations are performed with periodic boundary conditions, and a time step of 1.5 femtoseconds is adopted. For the production runs, the Nosé–Hoover thermostat and the semi-isotropic Parrinello–Rahman barostat are employed for temperature and pressure regulation, respectively. It is necessary to mention that the Berendsen thermostat at a small value of τ (e.g. τ = 0.01 fs) is extremely efficient for relaxing a system to the target temperature. But once the system has reached equilibrium, τ should be increased to get a good equilibrium run, and it might be more important to probe a correct canonical ensemble. Therefore, the Nose–Hoover extended method is appropriate for the production run. A 1.4 nm cutoff is applied to treat van der Waals interactions, and the long-range electrostatic interactions are treated with the Particle Mesh Ewald (PME) method^45^.

### Metadynamics simulations details

In addition, well-tempered metadynamics simulations are carried out to obtain free energy surface (FES) using the Gromacs 2019.2 patched with the PLUMED version 2.5.2 plugin^46^ via sum_hills tools. For the investigated systems, the 3D FES landscape is explored as the function of distances between the center of masses (COMs) of the A and Hexakis (CVDOX–Hexakis = d1) and between Hexakis and DMA (CVDOX–DMA = d2) in neutral pH at 298, and 320 K, and acidic condition at 298 K. The initial height and width for Gaussian hills that are deposited every 500 time-steps with a bias factor of 15, set to 1.0 kJ/mol, and width of 0.25 A°. In each system, the simulation ran for 60 ns to reach the global minimum at the equilibrium state. The exact global minimum for each set of FES landscapes is determined and used as a basis to calculate the 2D curves of the free energy. In these cross-sections, once the drug is considered fixed in its global minimum, and COM of the DMA is moved with respect to the carrier. The same pattern is repeated to derive a 2D curve for the drug. Overall, three individual 3D landscapes, and six 2D curves are obtained and explored. More details about the well-tempered metadynamics simulations will be provided in the “Results and discussion” Section. Details of all simulated systems are listed in Table 1.

**Table 1:** Simulation details of studied DOX–polymer–Hexakis systems.

Systems	Box size (nm^3^)	DOX	Number of polymer monomers	Number of Hexakis monomers	Number of ions	Number of water molecules
PNIPAM /DOX/Hexakis	9.0 × 9.0 × 13.0	5	5	10	120	31,882
DMA/DOX/Hexakis	9.0 × 9.0 × 13.0	5	5	10	132	31,806
Am/DOX/Hexakis	9.0 × 9.0 × 13.0	5	5	10	124	31,862
PDMA/PDOX/Hexakis	9.0 × 9.0 × 13.0	5	5	10	175	31,771

## Results and discussions

### MD simulations

**Figure 2:**
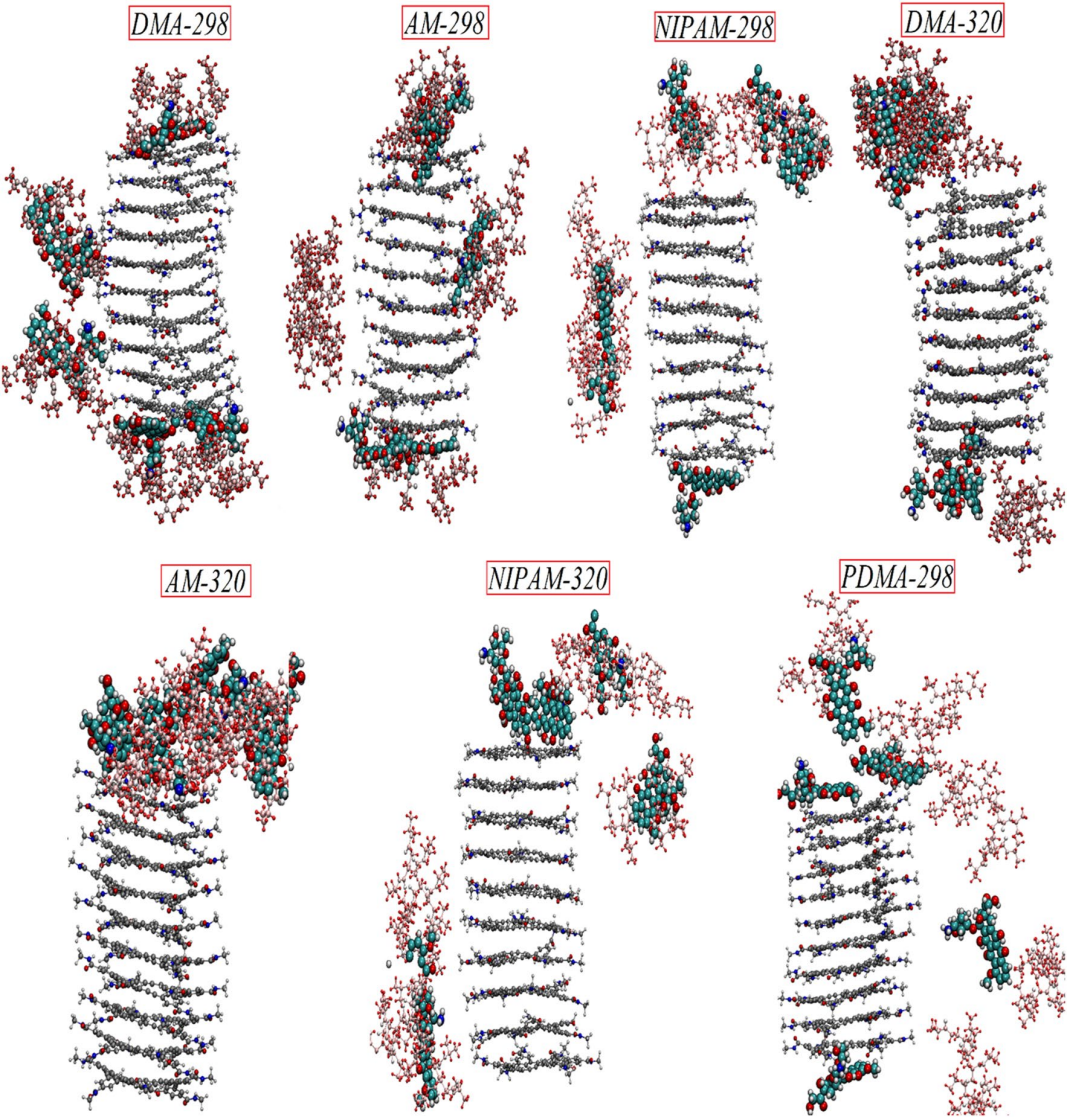
Snapshots of the loading systems after 60 ns MD simulation. Color code DOX: vdW cyan, Polymer: CPK violet, Hexakis: silver. The ions and water molecules are not shown for clarity.

To evaluate the role of PNIPAM polymer and two types of its copolymers in triggering the interaction between the DOX molecules and Hexakis nanotube, the MD simulations are performed at temperatures below and above LCST at 298 K, and 320 K, respectively. Since tumor cells have acidic pH, the DOX release mechanism from the DMA–Hexakis at acidic conditions also has been evaluated. Snapshots of the loading systems after 60 ns MD simulation are illustrated in Fig. 2. This Figure shows that the polymer chains at 298 K are approximately linear and interact with the DOX molecules, keeping them close to the carrier surface. While at 320 K, the polymer chains are in a globular state and surrounding the drug molecules and preventing them from interacting with the carrier surface directly, leading to DOX molecules overlapping together into the polymer chains. As can be clearly seen in Fig. 2, the Am/DMA copolymer chains have a more tendency to form complexes with the Hexakis and exhibit appropriate aggregation on the Hexakis nanocarrier surface than pure polymer. It is worthy to mention that these copolymers also demonstrate the controlled drug release at temperatures above the critical temperature. In addition, DMA chains are more stable and this will enable them to cover the DOX drugs better and form a more stable DOX–DMA–Hexakis structure at 298 K. In other words, all the DOX molecules are relatively stable all over the simulation in the presence of DMA chains and interact with the surface of the Hexakis nanotube through π–π stacking and hydrogen bonds.

#### Gyration radius

This finding can be confirmed by assessing the gyration radius (Rg) for the PNIPAM polymer and its two copolymers at 298 K and 320 K, respectively. The Rg is used to estimate the overall changes in the shape and compactness of the polymer structures in studied systems, which can be calculated using the following Equation:

1 $${R}_{g}^{2}=\left(\frac{{{\sum }_{i} {m}_{i}\parallel {r}_{i}\parallel }^{2}}{{\sum }_{i} {m}_{i}}\right)$$

where mi is the mass of the atom i, ri is the distance of atom i from the center of mass of the polymer. The gyration radius of PNIPAM–DOX–Hexakis, Am-DOX–Hexakis, DMA–DOX–Hexakis, and PDMA/PDOX/Hexakis simulation systems is shown in Fig. S1, Supporting Data. As shown in this Figure, the radius of gyration at 298 K is higher than Rg at 320 K, indicating that the PNIPAM polymer and its two copolymers are more compact above LCST concerning the 298 K. The dynamics of the gyration radius at 298 K revealed similar lengths during the simulation time and begin with Rg = 4.09 nm. After about 10 ns of simulations, the Rg at 320 K started to decrease the linearity to about 4.07 and 4.06 nm, while it is depicted more extended and flexible at 298 K (~ 4.10 nm). Indeed, the PNIPAM and Am/DMA polymers include repeating units of hydrophilic and hydrophobic groups, which allows them to be converted to a water-soluble or insoluble order depending on the temperature. Accordingly, due to the conformational changes from the flexible coil to a globular state, PNIPAM polymers can play a significant role in drug release control and drug absorption. These results are consistent with previous experimental findings of PNIPAM polymers functionalized-GO surface [93, 94]. The assembly of PNIPAM on the GO carrier is enabled to induce a globular-state above LCST (310.7 K) as well as a coil-state below LCST (298 K). It should be noted that the lowest and highest significant reductions of Rg are recorded for DMA copolymer at 298 K and 320 K, respectively, indicating the highest accumulation of this polymer around drug molecules and nanocarrier surface and also has the highest drug release.

#### Van der Waals and electrostatic energy

**Figure 3:**
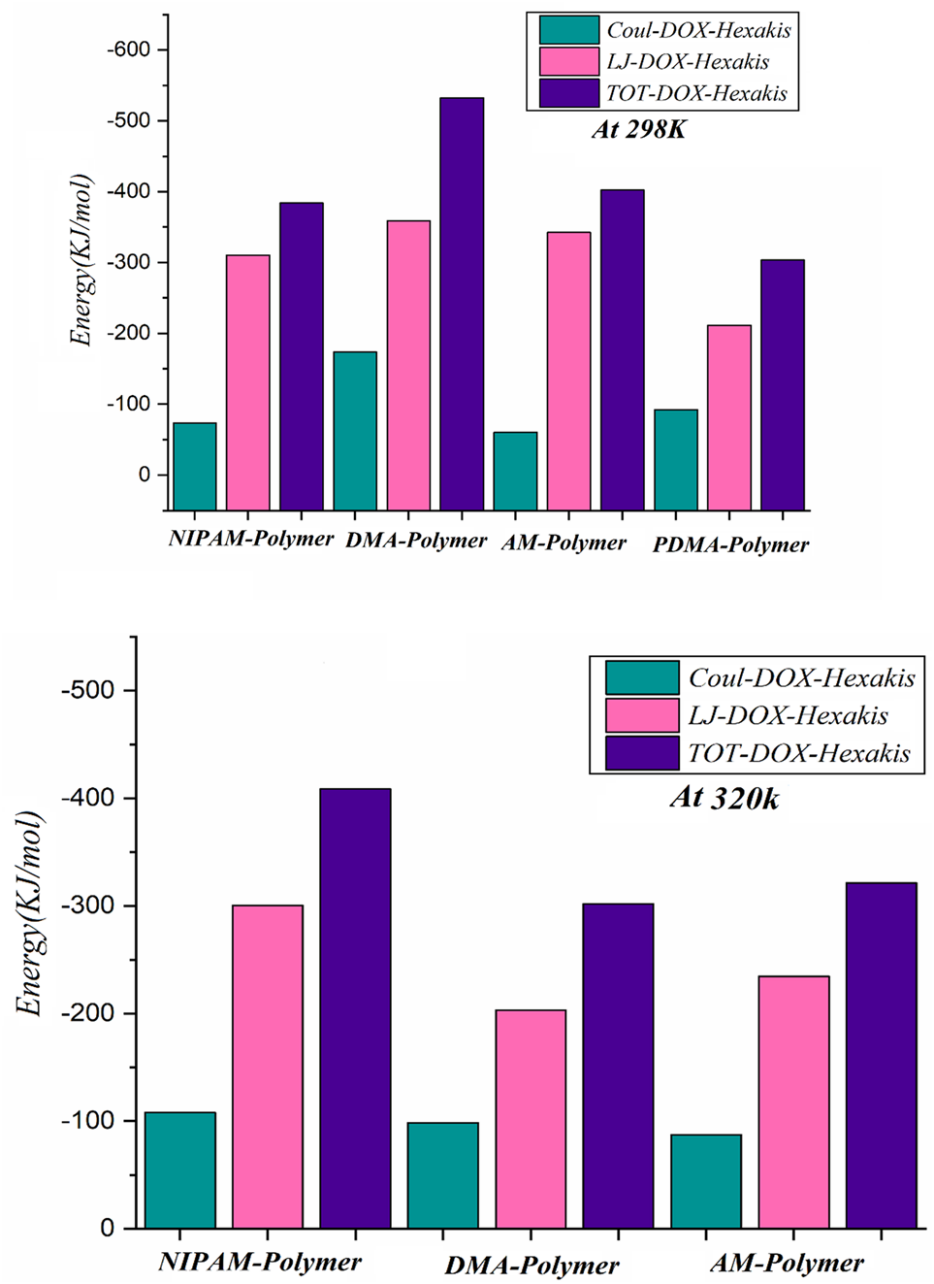
The average coulombic, van der Waals, and total interaction energies between different components of the studied systems.

Since the non-covalent interactions take part in affecting the binding affinity, the interaction energies between different components of each system are subjected to analyses and reported in Table 2. The negative values of binding energy indicate the spontaneous nature of the adsorption process. In all studied systems, the adsorption of drug molecules on the Hexakis surface mainly arises from vdW interactions, which can be attributed to the π–π stacking interactions between the aromatic groups of DOX and the Hexakis rings as seen in Table 2. The averaged interaction energies for the last 10 ns of each of the seven MD simulations are also projected in Fig. 3.

**Table 2:** A summary of the characteristics of the amino acids is explored in this investigation.

System	Interaction	At 298 K		At 320 K	
		L–J	Elec	L–J	Elec
PNIPAM /DOX/Hexakis	DOX–Hexakis	− 314.55	− 125.65	− 300.70	− 108.01
	DOX–PNIPAM	− 325.44	− 119.66	− 314.61	− 190.87
	PNIPAM–Hexakis	− 254.98	− 53.99	− 347.61	− 65.05
DMA/DOX/Hexakis	DOX–Hexakis	− 338.69	− 173.67	− 203.14	− 98.62
	DOX–DMA	− 334.03	− 151.76	− 312.34	− 36.73
	DMA–Hexakis	− 434.40	− 116.05	− 356.09	− 78.56
Am/DOX/Hexakis	DOX–Hexakis	− 242.38	− 28.87	− 234.54	− 87.33
	DOX–Am	− 487.34	− 261.71	− 383.099	− 108.26
	Am–Hexakis	− 304.01	− 154.88	− 148.104	− 60.39
PDMA/PDOX/Hexakis	PDOX–Hexakis	− 211.25	− 92.25		
	PDOX–PDMA	− 80.51	− 32.70		
	PDMA–Hexakis	− 6.59	− 1.08

This Figure reveals that the total interaction energies for all of the investigated systems are lower at 320 K compared to 298 K, suggesting a stronger interaction between the DOX molecules and Hexakis nanotube at 298 K. It can be understood due to the more binding region between the DOX and Hexakis surface stimulated by the tunable surface of PNIPAM when it is on the coil state at a temperature lower than LCST. According to the results, the values of vdW energies for DOX in DOX/DMA/Hexakis are − 338.69, − 203.14, and − 211.25 kJ/mol at T = 298, 320 K, and protonated system, respectively. Such results could be explained by considering different contributions; (i) with increasing temperature; the interaction between DMA and Hexakis surface is decreased, as a consequence, the structure of the polymer change to a globar state at high temperature, and minimizing the DOX interacting with DMA and carrier surface is occurring through both electrostatic and VdW interactions. (ii) Under the protonate condition, the CNH-groups of DMA and DOX provide repulsive interactions between themselves as well as with the nanocarrier surface, but the rate of repulsion is much lower than with increasing temperature. Therefore, it is concluding that increasing the temperature of polymers is an important factor in drug release. However, the PDMA could respond to acidic conditions by protonation and followed by increased hydrophilicity and swelling of the polymer structure, resulting in the drug being released. In addition, the pH- or temperature drug release profiles suggest that the PNIPAM and two of its copolymers that loaded on Hexakis nanocarrier facilitated controlled release in response to the different simulated microenvironments. Temperature-dependent release studies from DOX–DMA–Hexakis indicated a slower release of drugs below LCST and a sustained release above LCST. Because the side chains of copolymers have more hydrophobic methyl (−CH_3_) groups, these polymers underwent the most serious reduction of the interaction with water molecules. For this reason, the interactions between DMA/Am copolymers and water molecules are less than PNIPAM and interact more with DOX molecules. From this result, it can be inferred that such a different deswelling process of PNIPAM from that of its copolymers in our simulations validates the soundness of the simulation methods.

#### The number of hydrogen bonds

The hydrogen bond (HB) between two atoms is defined as a pair of donor–acceptor with an angle smaller than 30°. The number of polymers–water, and polymer–DOX hydrogen bonds is shown in Fig. S2 for the three simulations below and above the LCST. As mentioned above, the structure of PNIPAM polymer and its copolymer consists of repeating units of hydrophilic and hydrophobic groups, which allows them to become a water-soluble or insoluble order depending on the temperature and have transformation from the coil to a globular state. As Fig. S2 suggests, PNIPAM polymer will result in a higher number of hydrogen bonds formed between solvent and polymer, while this polymer exhibited the least number of HB with DOX molecules. In contrast, the formation of HB between DMA/Am copolymers and solvent is less than PNIPAM and has more HB with DOX molecules. Because the side chains of copolymers have more hydrophobic methyl (–CH_3_) groups, these polymers underwent the most serious reduction of the interaction with water molecules. In addition, the increase in the number of hydrogen bonds between water molecules and polymer chains is observed above the LCST, indicative of higher hydrophilicity at 320 K, better polymers dispersion in water, more stability, and probably less aggregation around the Hexakis nanotube. As the formation of the globular phase above the LCST is observed in the simulation process. This finding suggests that hydrogen bonding between polymer and water plays a very important role in maintaining the solvation and keeping the polymer hydrophilic, especially above the LCST.

#### Radial distribution function

The radial distribution function (RDF) profile is a suitable tool for describing the distribution of a guest molecules around a host surface. It is another way to find the adsorption geometry of the ligand on the substrate that determines the probability of finding particles i around particles j at a function of distance (r) which is calculated based on the following equation:

2 $$\mathrm{g }(\mathrm{r}) =\frac{1}{{{N}_{i}\rho }_{j}} \sum_{i}^{{N}_{i}}\sum_{j}^{Nj}\frac{\delta ({r}_{ij}-r)}{4\pi {r}^{2}}$$

**Figure 4:**
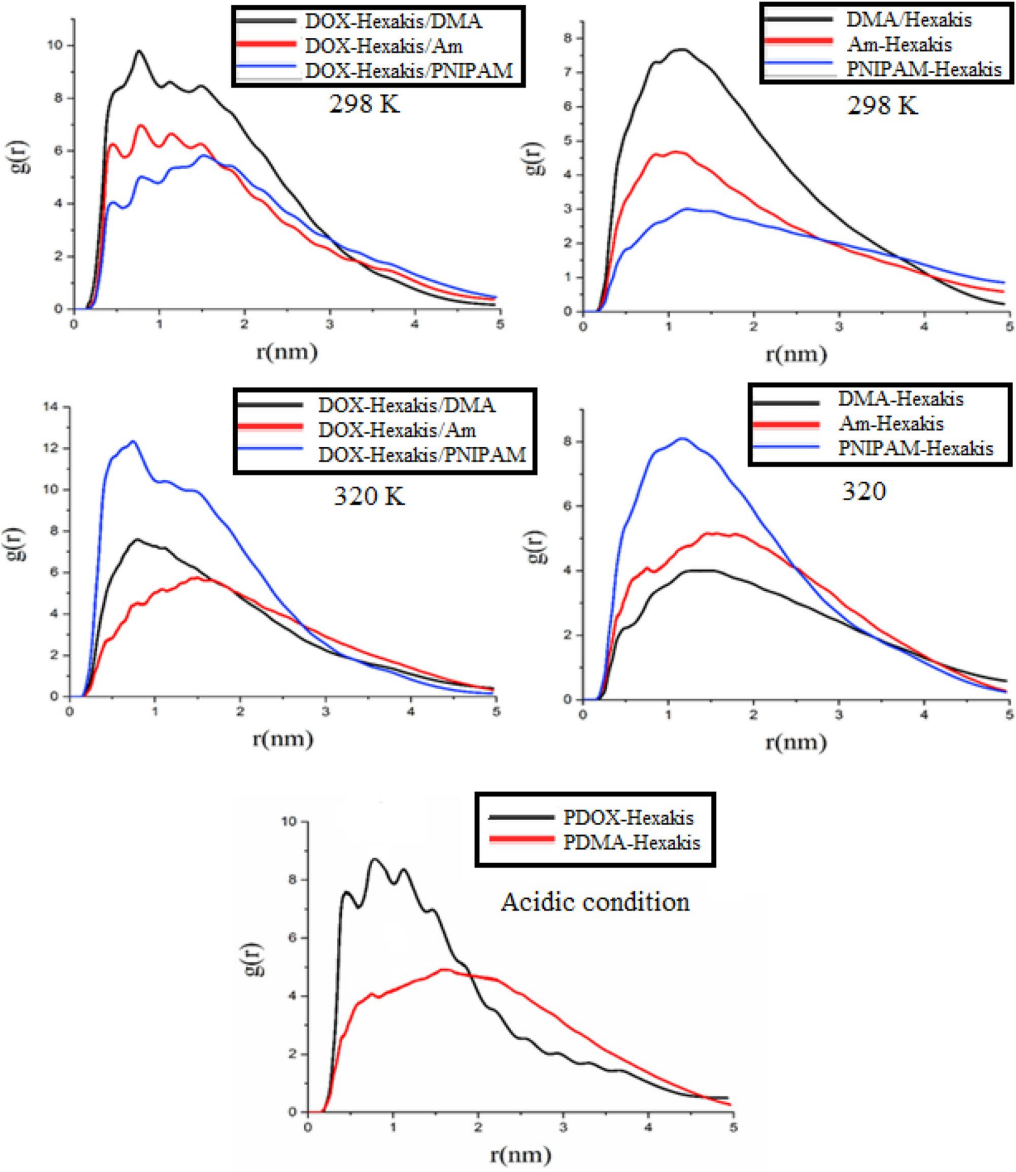
Radial distribution function of the DOX and polymer molcules around the Hexakis surface in the study systems.

The RDF patterns of different components of the studied systems and also between active sites of DOX (aromatic, glycol, and amine parts) and DMA polymer chains are presented in Fig. 4. At 298 K, the highest accumulations of DOX and DMA, which have stronger interactions with the substrate, are located at 0.1 to 0.8 nm away from the Hexakis surface, while the most probable distance for DOX and Am/PNIPAM to the Hexakis are observed at about 0.7–0.5 and 0.8–0.6 nm, respectively, (see in Fig. 4). This result indicates that after equilibration, all of the DOX molecules are positioned on the surface of Hexakis in the DOX–DMA–Hexakis system because the DMA polymer covered the DOX molecules more completely than those others did. In other words, these results showed the stronger and more compressed adhesion of DOX molecules with DMA as compared to other polymers. A close inspection of Fig. 4 reveals that in 320 K, the probability of finding drugs and polymers is lower both in terms of closeness to the surface and amount. This is in agreement with the fact that the total energies and L–J for all study systems at 320 K are less than 298 K, and in the high temperature the polymer chains change to a global state, and approximately the drugs are released. As well as, Fig. 4c reveals that in acidic conditions, DOX is closer to the carrier surface but is present in less amount, and a higher amount of polymers accumulated a little farther away from the carrier.

Generally, structural analysis performed using various dynamical correlation functions, such as radius of gyration, radial distribution function (RDF), and HB suggests that the Hexakis–DMA (system C) is the best carrier at temperatures blow and above LCST compared to two other systems.

### Metadynamics simulations

**Figure 5:**
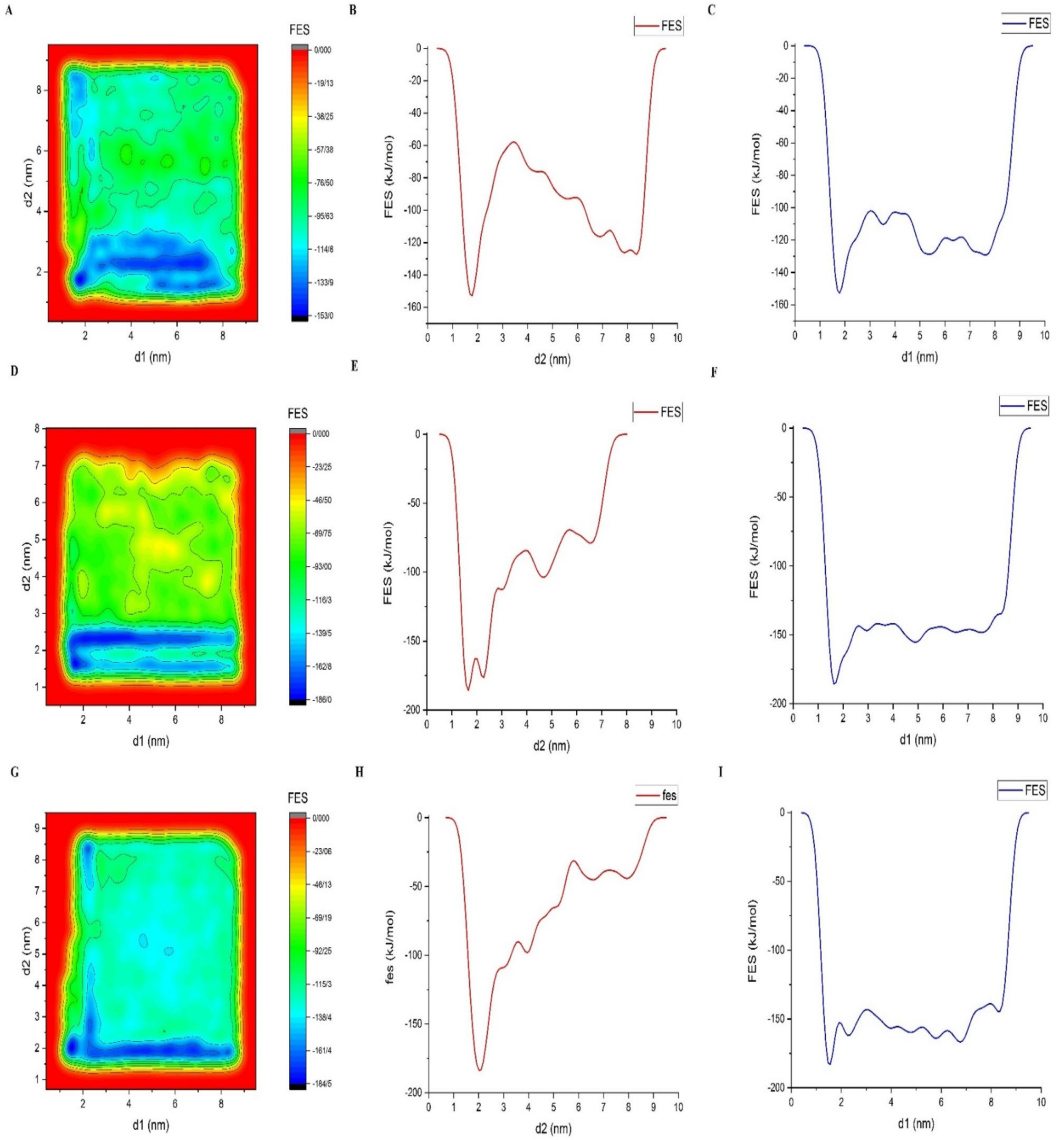
Left: Free energy landscape as a function of d1 and d2, which are the distance of the center of mass of DOX from the center of mass of Hexakis nanotube and DMA polymer chain, Middle: Free energy surface *for* Hexakis and different orientations of DOX, Right: Free energy surface for Hexakis and different orientations of DMA polymer chain, respectively.

In order to explore the free energy landscape for the process of simultaneous adsorption/release of DOX and DMA on the Hexakis surface, three different series of well-tempered metadynamics are carried out. First, FES is explored for the competitive adsorption of DOX alongside DMA on the surface of Hexakis nanotube at 298 K. Then, the same process is repeated at 320 K, and lastly, the final configuration of the first series is extracted and protonated to be used as the initial materials of the desorption process at 298 K (Fig. 5). As can be seen in Fig. 5, panel A, the global minimum is reached at distances of d1 = 1.63, d2 = 1.66 nm with − 185.74 kJ/mol free energy. While the same process is repeated at 320 K, the free energy of the global minimum collapsed to − 152.82 at a little longer distance (d1 = 1.78, d2 = 1.78 nm). These results can be attributed to the thermo-sensitive nature of the DMA, which leads to a decrease in adsorption free energy and also facilitates the release of the drug. To some extent, this feature is related to the presence of methyl groups in the structure of DMA. These hydrophobic functional groups tend to attract each other at T > LCST.

In order to further explore the behavior of the complex in a carcinogenic environment, the protonated structure of the final configuration of the absorption process at 298 K, are subject to metadynamics simulation for 60 ns. Results showed a limited reduction in the free energy of the adsorption complex (− 183.06 kJ/mol compared to − 185.74 kJ/mol for adsorption). Nevertheless, the coordination of the molecules’ COM changed noticeably with respect to Hexakis. In fact, protonation leads to a slight decrease in the distance of DMA with respect to Hexakis (d1 = 1.55 compare to d1 = 1.63 nm for the adsorption form). This might be a result of an improvement in DMA adsorption to the Hexakis, which is solely revealed by Metadynamics simulation. However, these simulations in an acidic environment show an increase in the distance of the DOX molecule from the Hexakis (d2 = 2.1), which may suggest the facilitation of the release of the drug.

Another way to look at the FES landscape is to assess the effects that the orders of insertion of molecules might have on the free energy curves of the explored processes. Considering that the DMA is absorbed first and then the drug is inserted as the second molecule, the free energy of the system will gradually become more negative with a decrease in distance. It is worth mentioning that in the higher temperature conditions, there is an energy barrier with a height of around 80 kJ/mol that on one hand, deter the absorbed molecule from being fully detached from the carrier. On the other hand, fluctuations in the energy of the system are high enough to give the molecule a chance to slowly pass this barrier. This is also proof of the thermo-sensitive property of the explored polymers.

However, if DMA is inserted into the system as the second molecule while DOX is already absorbed, changes in the position of the polymer will lead to a limited variation in the Free energy surface. In fact, absorption of DMA in such a situation will lead to a 30 kJ/mol reduction in free energy of the system. It seems that in a situation where DOX is absorbed on Hexakis in advance, insertion of DMA will lead to an unnoticeable change in the free energy of the system. In other words, simultaneous insertion of both molecules will lead to better results, both in terms of adsorption and desorption processes.

## Conclusion

The purpose of this study is to design and evaluate a new delivery vehicle based on the Hexakis–polymer composite and to explore its potential application as an innovative drug delivery system. For the first time, the Hexakis–polymer composites as dual pH- and thermo-sensitive delivery vehicles for the anticancer drug DOX are examined using the MD and metadynamics simulations method. Therefore, in order to study the effect of thermosensitive polymers on the DOX delivery system, three different types of carriers are considered; Hexakis–PNIPAM, Hexakis–Am, and Hexakis–DMA. The results of this study revealed that the Hexakis–polymer carrier could be a suitable carrier for the adsorption and release of Doxorubicin. Interestingly, 10-mer polymer chain lengths of PNIPAM and its copolymers are found to be a suitable for DOX-loaded drug carriers based on the dynamical results of this work.

The obtained interaction energies revealed that the DOX molecules have lower interaction with the Hexakis–polymer carrier at 320 K compared to 298 K, and this finding is confirmed by the Rg and RDF analyses. The study of gyration radius showed that the most considerable reduction in gyration radius occurred at 320 K than 298 K, which is indicative of the most compressed polymer molecules aggregation above LCST. Moreover, the highest significant reductions of Rg are recorded for DMA copolymer, indicating the highest accumulation of this polymer around drug molecules and nanocarrier surface and also has the highest drug release. Radial distribution function analysis showed the stronger and more compressed adhesion of DOX molecules with DMA because the DMA polymer covered the DOX molecules more completely than those other polymers. Also, this study sought to improve the properties of the Hexakis nanotubes for Doxorubicin release. Therefore, the pH-responsive adsorption of DMA onto the Hexakis is studied to examine the role of protonation at 298 K. It is found that DMA, which has titratable side chain groups –N (CH_3_)_2_, is responsive to pH changes by protonation of the side-chain moieties. Obtained results concludes that neutral pH is favorable for the adsorption of DOX on the Hexakis–polymer composite, and at acidic pH, the drug releases from the surface of the carrier. The well-tempered metadynamics simulation results showed that the order of insertion of drug and polymer into the system plays a crucial role in determining the fate of the adsorption/desorption process. In this content, DMA composite with Hexakis is a better carrier for drug delivery. Overall, our MD and metadynamics simulations revealed that such behavior of polymer–Hexakis composite makes it a promising candidate for the development of a wide range of new generations of intelligent DDS. As a recommendation for further studies, Hexakis nanotube can be used as drug carriers along with other compounds, especially polymer components. It is necessary to take further steps to improve the properties of new Hexakis nanotubes which can be utilized on a commercial scale.

## Supplementary Information

Supplementary Figures.

## Data Availability

Authors can confirm that all relevant data are included in the article and/or its supplementary information files.
