# Author Correction: PNIPAM/Hexakis as a thermosensitive drug delivery system for biomedical and pharmaceutical applications

**DOI:** 10.1038/s41598-022-20711-9

**Published:** 2022-09-27

**Authors:** Samaneh Pasban, Heidar Raissi

**Affiliations:** Department of Chemistry, University of Birjand, Birjand, Iran

Correction to: *Scientific Reports* 10.1038/s41598-022-18459-3, published online 23 August 2022

The original version of this Article contained an error in the order of the author names, which was incorrectly given as Heidar Raissi & Samaneh Pasban.

The original Article has been corrected.

