# Supplementary material for: PNIPAM/Hexakis as a thermosensitive drug delivery system for biomedical and pharmaceutical applications
Source: Sci Rep. 2022 Aug 23;12:14363. doi: 10.1038/s41598-022-18459-3 (PMC9399122; doi:10.1038/s41598-022-18459-3)
Supplement: Supplementary file 1 — Supplementary Figures. [file 41598_2022_18459_MOESM1_ESM.docx]

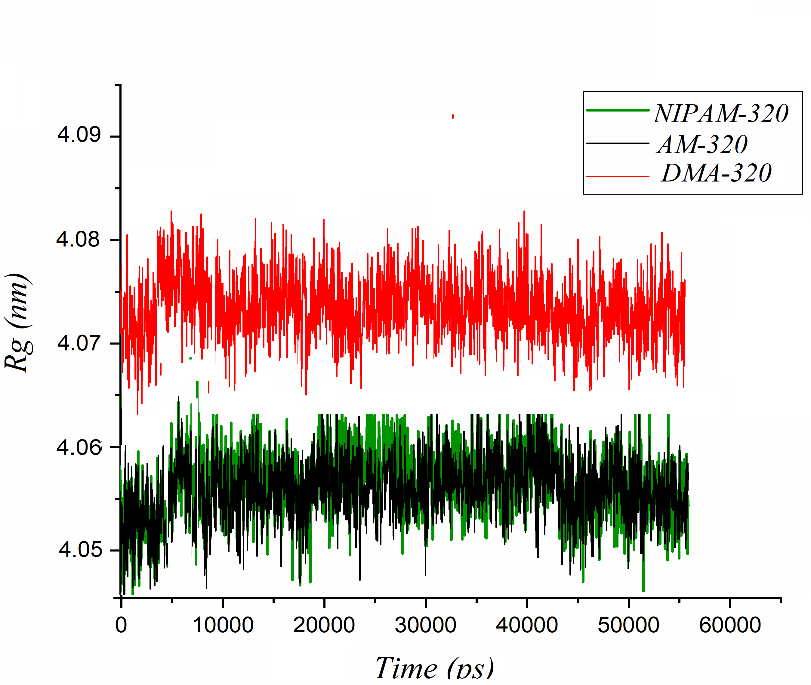

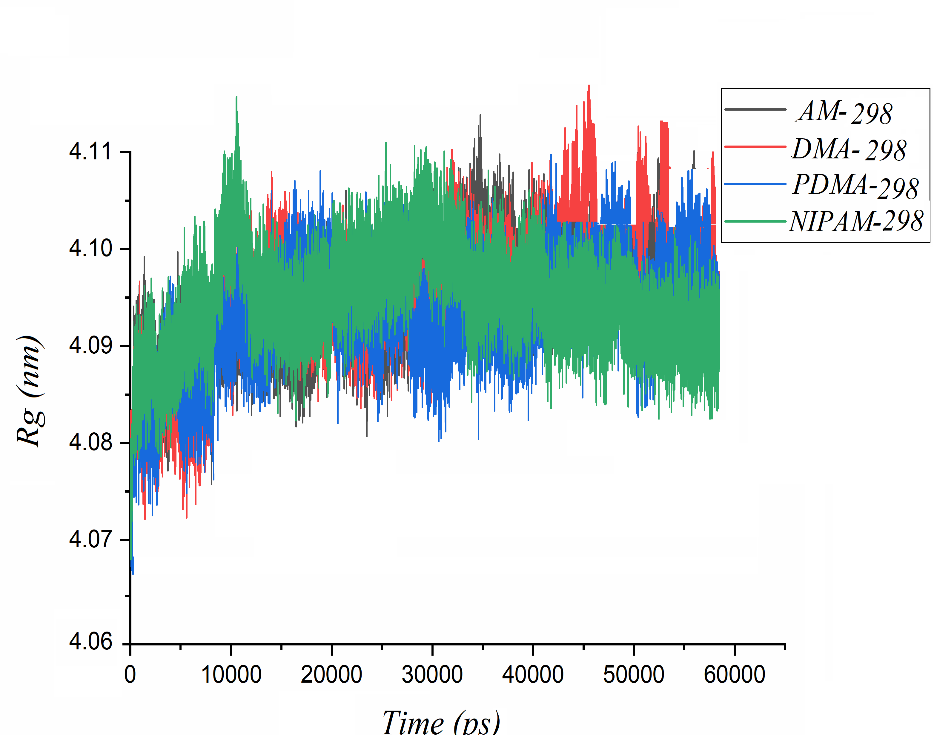


**Figure S1:** The gyration radius of the simulation systems

**
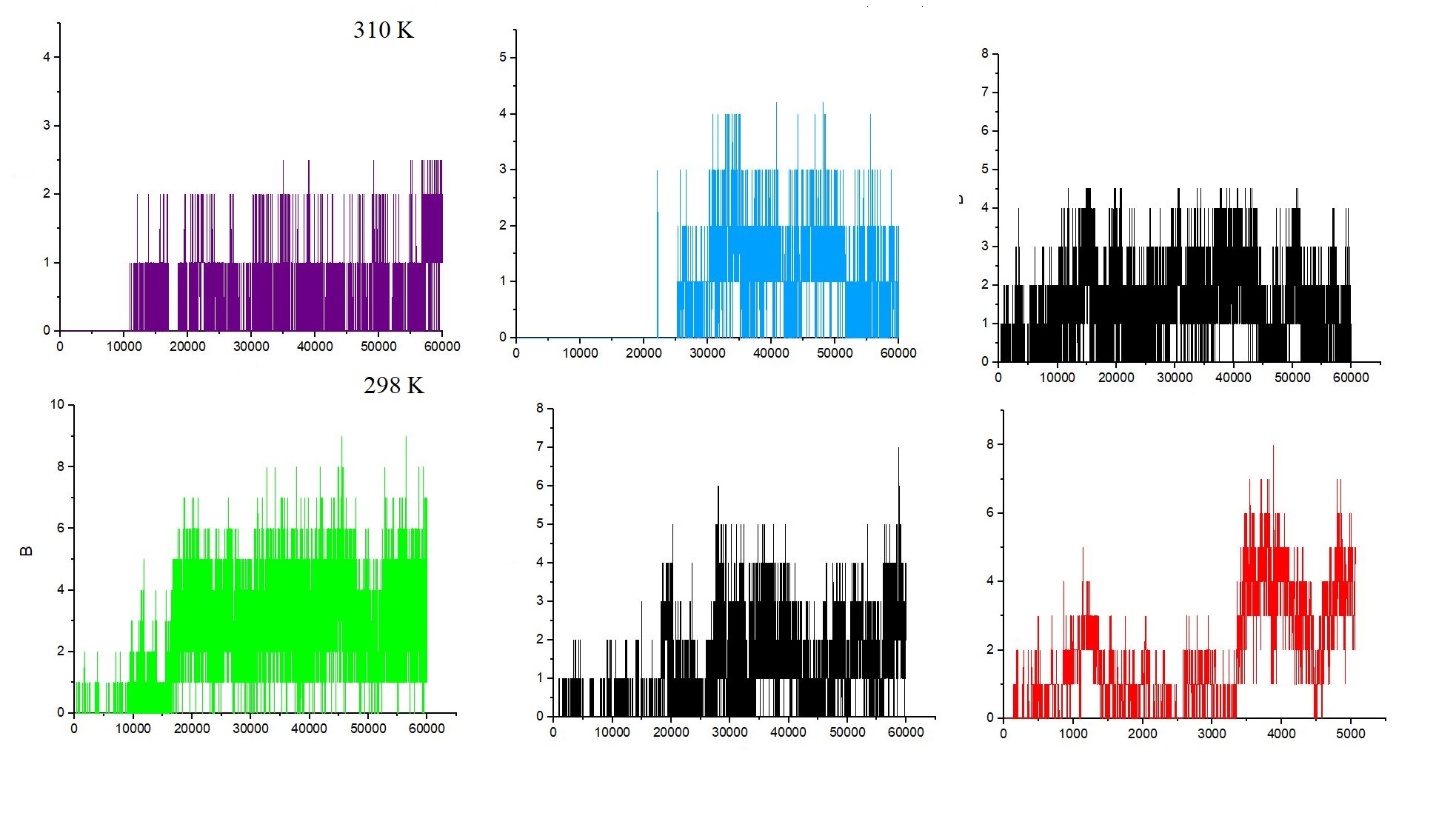
**

**Figure S2:** The number of HB between DOX and polymer of the simulation systems
